# Stops making sense: translational trade-offs and stop codon reassignment

**DOI:** 10.1186/1471-2148-11-227

**Published:** 2011-07-29

**Authors:** Louise J Johnson, James A Cotton, Conrad P Lichtenstein, Greg S Elgar, Richard A Nichols, p David Polly, Steven C Le Comber

**Affiliations:** 1 School of Biological Sciences, University of Reading, Reading, UK; 2 School of Biological and Chemical Sciences, Queen Mary, University of London, London, UK; 3 Wellcome Trust Sanger Institute, Hinxton, Cambridge, UK; 4 Population Genetics Technologies Ltd, Minerva Building, Babraham Research Campus, Cambridge, UK; 5 MRC National Institute for Medical Research, Mill Hill, London, UK; 6 Department of Geological Sciences, Indiana University, Bloomington, Indiana, USA

## Abstract

**Background:**

Efficient gene expression involves a trade-off between (i) premature termination of protein synthesis; and (ii) readthrough, where the ribosome fails to dissociate at the terminal stop. Sense codons that are similar in sequence to stop codons are more susceptible to nonsense mutation, and are also likely to be more susceptible to transcriptional or translational errors causing premature termination. We therefore expect this trade-off to be influenced by the number of stop codons in the genetic code. Although genetic codes are highly constrained, stop codon number appears to be their most volatile feature.

**Results:**

In the human genome, codons readily mutable to stops are underrepresented in coding sequences. We construct a simple mathematical model based on the relative likelihoods of premature termination and readthrough. When readthrough occurs, the resultant protein has a tail of amino acid residues incorrectly added to the C-terminus. Our results depend strongly on the number of stop codons in the genetic code. When the code has more stop codons, premature termination is relatively more likely, particularly for longer genes. When the code has fewer stop codons, the length of the tail added by readthrough will, on average, be longer, and thus more deleterious. Comparative analysis of taxa with a range of stop codon numbers suggests that genomes whose code includes more stop codons have shorter coding sequences.

**Conclusions:**

We suggest that the differing trade-offs presented by alternative genetic codes may result in differences in genome structure. More speculatively, multiple stop codons may mitigate readthrough, counteracting the disadvantage of a higher rate of nonsense mutation. This could help explain the puzzling overrepresentation of stop codons in the canonical genetic code and most variants.

## Background

Premature termination of protein synthesis is costly, whether it is caused by heritable mutation, transcriptional error or mistranslation. Many disease genes are nonsense mutations [e.g. [1,2]], and the existence of nonsense-mediated mRNA decay, a specialized mechanism to promote rapid degradation of mRNA containing premature translation termination signals, provides evidence that premature termination is also costly if the error occurs at the translational level [3]. Premature termination of translation is at best a waste of resources, and at worst can produce abnormal polypeptides that interfere with normal protein function.

Similarly, readthrough - a failure to terminate protein production at the stop codon -appears to be selected against. Both prokaryotes and eukaryotes use conserved release factors to recognise stop codons and ensure the prompt release of ribosomes [4,5], and the occurrence of termination sequences in *Saccharomyces cerevisiae *is negatively correlated with readthrough [6]. The existence of 'tandem stops', in-frame secondary stop codons which are conserved between species [7] and correspond to variations in the genetic code [8] also suggests that selection acts at the sequence level to ameliorate readthrough (but see [9]).

The relative frequency and cost of these two types of error depends on many factors, including the efficiency of the transcriptional termination machinery and the presence of conserved tandem stop codons. However, the genetic code could also have a strong influence. Regarding readthrough, a code with more stops increases the likelihood that a readthrough product will swiftly be terminated even in the absence of conserved tandem stops, as random downstream intergenic sequence will contain frequent stop codons by chance. Regarding premature termination, each stop codon in the genetic code is associated with a number of error-prone "near-stop" codons that are mutationally adjacent: nine other triplets are each a single point mutation away from any one stop codon.

Such codons are prone to deleterious point mutation, and this is likely to hold true for transcriptional and translational errors also, as all three processes rely on base pairing. A selective disadvantage to near-stop codons might therefore be visible in genome sequences, perhaps manifesting as, or mediated by, codon usage bias. Codon usage bias varies between organisms, between genes, and, in some cases, along genes [10], and can be due to selection for translational speed or accuracy, or to mutational biases [11]. Mutational biases, and the tRNA abundances that mediate selection for transcriptional efficiency, are themselves under some degree of genomic control, so selection could affect codon usage bias through either of these conduits as well as acting directly on DNA sequences.

The strongest influence on preferred codon identity in most species is GC content [12], but there are other patterns: where synonymous codons can end in T or A, T is generally favoured. This has the effect of creating fewer of the near-stops TCA, CGA and GGA.

### Model and Tests

Below, we will discuss our model in terms of mutations to the DNA sequence during replication, rather than translational or transcriptional errors. However, base pair substitutions in transcription, or single base pair misinterpretations in translation, will follow the same pattern. We therefore expect the same selective forces to be at work in all three processes.

We assume that an organism whose genetic code contains S stop codons is subject to a total cost, *C_S_*, given by the sum of the cost of premature termination, *C*[τ]*_S_*, and the cost of reading through a coding sequence's terminal stop, *C*[ρ]*_S_*; that is, that *C_S _*= *C*[τ]*_S _*+ *C*[ρ]*_S_*.

For a coding sequence of *N *triplets, any mutation must occur in either one of *N*-1 amino acid-encoding triplets, or the terminal stop triplet. For a genetic code with *S *stop codons, *C*[τ]*_S_*, the total cost of premature termination is given by the probability that a mutation will occur in an amino acid-encoding triplet, the likelihood that such a mutation will produce a stop and the cost per sequence:(1)

where π [aa:stop] is the proportion of point mutations that will alter an aa-encoding triplet to a stop triplet and τ is the cost per sequence of premature termination. *k *is a constant discounting the cost of premature termination, to take into account mechanisms of cost reduction such as nonsense-mediated mRNA decay or a selectively maintained deficit of near-stops in coding sequences.

The total cost of reading through the coding sequence's terminal stop, *C*[ρ] *_S_*, is given by(2)

where ρ is the cost per sequence of reading through the coding sequence's terminal stop, π[stop == > aa] is the proportion of non-synonymous point mutations to the coding sequence's terminal stop and *R *is the maximum number of amino acid residues that can be appended to the protein's C-terminus without affecting the protein's function. That is, the total cost is the product of the probability that a mutation affects the terminal stop, the proportion of such mutations that will alter the stop to a triplet encoding an amino acid (corrected for the proportion of sequences rescued by the presence of an in-frame stop downstream of the correct stop) and the cost per sequence. Each of these processes - premature termination and readthrough - will increase with mutation rate; however, the model relies on the relative probabilities of these two processes, not their absolute probabilities.

Since by definition *C*[ρ]*_S _*excludes cases in which protein function is rescued by the presence of a downstream stop within *R *residues, for any given sequence we take the costs of premature termination and readthrough to be equal, since in each case the resulting protein is non-functional; that is, we set τ = ρ = 1.

The parameters π[aa:stop] and π[stop:aa] in our model vary with genetic code: crucially, changes in stop codon number have opposite effects on the probability of premature termination and readthrough, in that more stop codons make premature termination more likely, but readthrough less likely. Both parameters are also affected by the pattern in which stop codons are added to the genetic code. Here, we consider cases in which stop codons occur in blocks; that is, a new stop codon will be mutationally adjacent to existing stop codons. This is not the case for all known sets of stop codons, but is conservative for our purposes, as it ensures that as *S *increases, the increase in π [aa:stop] is minimised. The values of π [aa:stop] and π [stop:aa] under this assumption, considering only single base pair substitutions, are shown in Table 1.

**Table 1:** Values of π[aa:stop] and π[stop:aa] as number of stop codons, S, increases

*S*	**π [aa**⇒**stop]**	**π [stop**⇒**aa]**
1	1/63	1
2	8/279	8/9
3	7/183	7/9
4	2/45	2/3
5	31/531	31/45
6	2/29	2/3
7	13/171	13/21
8	5/63	5/9
9	1/11	5/9
		

## Results

### Codons mutationally adjacent to stops are underrepresented in human genes

**Figure 1:**
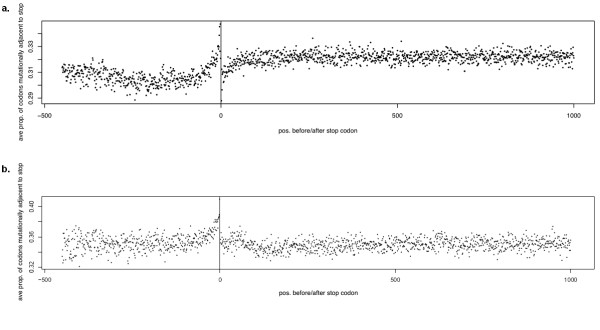
**Proportion near-stop codons in human coding sequences**. The proportion of triplets within a single point mutation of a stop codon, as a function of distance from the correct stop, for 500 bp upstream and 1000 bp downstream of a) all protein-coding transcripts in the human genome; b) all coding sequences in the yeast genome. Negative values represent upstream triplets and positive values downstream triplets.

We calculated the proportion of near-stops - triplets that are mutationally adjacent to a stop codon - as a function of distance from the correct termination codon for all human and, for comparison, yeast genes (Figure 1). We interpret the results as follows.

In humans, near-stops are underrepresented in coding sequences as compared to the downstream region. This was tested by randomisation, which accounts for the differing AT content of genes and downstream regions (see Methods).

Secondly, the proportion of near-stops initially declines along the coding sequence (confirmed by linear regression). Codon usage bias is seen to increase 5'-3' along coding regions in several genomes, perhaps because accelerations in translation rate can prevent ribosomes from colliding. However, codon preference in humans is thought to be largely driven by mutational pressures rather than selection for translational efficiency [13] but see[14]. We suggest that this decline occurs because premature truncation, where it destroys protein function, wastes more resources the later in translation it occurs, and therefore near-stops are more strongly disfavoured if they occur late in the coding sequence.

Thirdly, the proportion of near-stops rises sharply just upstream of the true stop codon. This spike may be partly due to a relaxation of selection when truncation happens close enough to the true stop to allow protein function, but as it rises above the level of noncoding DNA, we suggest it represents past readthrough mutations which have recently elongated coding regions. The dip immediately downstream from the stop codon is also interesting. One possibility is that this represents a region in which selection has converted a proportion of the available near-stops into tandem stop codons, although these have not been directly detected in human sequence data.

In yeast, however, neither the overall deficit of stop codons in coding as compared to non-coding sequence, nor the decline along the coding sequence, are seen (the presence of the declines was tested by linear regression within the coding sequence, truncated just before the spike). The peak just upstream of the true stop remains. The more pronounced difference in AT richness between yeast genes and intergenic regions may explain the first difference. We suggest that the decline is non-existent in yeast because translational selection is stronger and near-stops are not tolerated even at the 5' end of coding sequences.

It appears, then, that in some organisms there is evidence of selection at the DNA sequence level to avoid near-stops in coding sequences, as well as to accumulate tandem stops downstream of them. These are tasks which organisms with differing stop codon numbers will be differently competent to accomplish.

### Length of coding sequences declines with stop codon number

Our model also predicts that genetic codes could influence the length of coding sequences. In organisms whose code contains fewer stop codons, one constraint on coding sequence length is loosened, as preventing readthrough becomes relatively less important compared to preventing premature termination.

**Figure 2:**
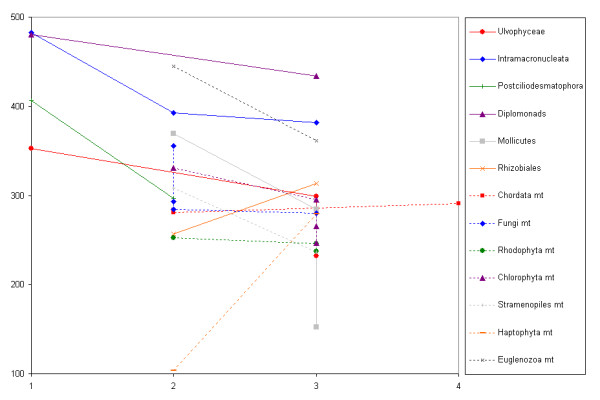
**Length of coding sequences**. Average gene length (y-axis) of GenBank coding sequences from taxa within which genetic codes differ in stop codon number (x-axis). Solid lines, nuclear genes; dotted lines, mitochondrial genes. See Methods for details.

For 13 taxa whose members differ in stop codon number, Figure 2 shows the average length of coding sequences plotted against stop codon number (see Methods for details). These groups have certainly been unequally sampled, there are some groups from which few sequences are available, and many taxa will have been incorrectly assigned to the universal code simply because contrary evidence has never been sought. These considerations will add noise, but should not bias our results. Interpreted conservatively, 14 changes in stop codon number are observed (13 groups are shown, one of which involves at least 2 code changes), of which 11 are in the direction of increased coding sequence length with fewer stop codons. This gives a p-value of p = 0.057 in a two-tailed sign test: inconclusive, but consistent with our hypothesis. Note that the sign test does not require that each individual difference also be statistically significant: nevertheless, significant differences are highlighted in Additional file 1 Table S1, which also gives details of the sequences used in this analysis.

### Truncations or gene loss?

Truncation mutations will occur less often if there are fewer stop codons, while readthrough mutations will extend proteins further if there are fewer stop codons. These two statements are central to our argument, but also present an alternative explanation for a negative correlation between stop codon number and gene length.

By aligning homologous genes from related organisms with differing genetic codes, it is possible to discern whether changes in gene length are due to simple truncation or extension at the 3' end of genes, indel mutations throughout the length of genes, or differences in genome composition (e.g. a disproportionate loss of shorter genes corresponding to stop codon reassignment). The mollicutes *Mycoplasma agalactidae *(NC_013948; 1 Mb; 812 coding sequences of average length 361 amino acids; 2 stop codons) and *Acholeplasma laidlawii*, (1.5 Mb; 1379 coding sequences of average length 326 amino acids; 3 stop codons) were chosen for this analysis. These have comparable overall genome sizes and show a significant difference in gene length (p < 0.01; t-test assuming unequal variance). 103 pairs of homologues were identified by enzyme name; these were likely to be highly conserved, for better alignment. Of these, the *M. agalactidae *homologue was longer in 56 instances (not in itself a significant difference). If changes in gene length were solely due to different rates of truncation or extension mutations, then after removing any 3' unaligned residues the genes should be of equal length. This was not the case: of the 56, the Mycoplasma homologue was still longer in 49 cases, a significant majority (p < 0.001, binomial test).

## Discussion

### Avoidance of near-stops in human coding sequences

The underrepresentation of near-stops in human genes is not due to AT content. However, it could be the result of codon bias, or - more interestingly - a disadvantage to near-stops in coding sequences could be a selective explanation for aspects of codon bias. Further analyses will be needed to clarify causal relationships and to help explain the differences between yeast and human genomes. Population genetic modelling would also be valuable, to work out how strong selective forces need to be and whether the potential for genomic mutation, or for transcriptional and translational error, is more likely to result in near-stop avoidance.

### Influences of genetic codes on genome structure

Intriguingly, a relationship between overall genome size and codon reassignment has been noted before, but in the opposite direction to that suggested by our model: TGA is frequently reassigned to a sense codon in small genomes [15,16], including mitochondrial genomes, for which there is a higher possibility that any one stop codon will drift to complete loss. We would suggest that stop-to-sense codon reassignment corresponds with decreasing gene number, but increasing gene length. Where there is strong selection for genome reduction, there will be downward pressure on both gene length and number: in such a situation, the balance of selective and mutational pressures would be extremely complicated.

### AT content and codon reassignment

AT-rich genomes are particularly prone to stop codon reassignment [17], but see [16]. This has been attributed to codon capture, a process by which a stop codon drifts to complete disuse and can then be reassigned in a selectively neutral manner. However, AT-richness also tips the balance of costs toward premature termination of translation: AT rich genomes will have more near-stops in their coding regions, and chance alone will generate plentiful tandem stops. Our model therefore explains the fact that while stop codons are indeed frequently lost from AT-rich genomes, the stop codons UAG and UGA - which are more likely than UAA to drift into disuse in AT-rich genomes- are not themselves disproportionately prone to reassignment [16].

### An adaptation of the code itself?

The genetic code is not arbitrary. The canonical genetic code shows good evidence of selection to minimise the effect of errors [18], and to allow transcripts to contain many simultaneous messages, including protein binding sites, splicing signals and RNA secondary structural motifs [19]. Constraints on code evolution are extremely strong, because a change in the translation table alters many gene products simultaneously. However, there is strong evidence that natural selection can occasionally bring about just such drastic changes. Some extant variation in genetic codes appears to be adaptive: codon reassignments in mitochondria, for example, occur in response to selective pressures on the amino acid composition of proteins [20]. Note that selection for the same trait often acts at both levels: for example, error minimisation is built in to the code, but there is also strong conventional selection on polymerase genes for copying fidelity. If we allow that genetic codes, despite being highly constrained, are or have been capable of adaptive evolution, the balance between readthrough and premature termination is another selective pressure that could influence, as well as respond to, changes in stop codon number.

**Figure 3:**
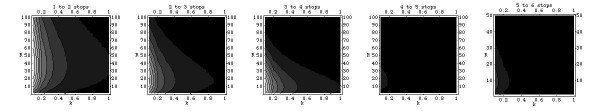
**Thresholds for changes in the number of stop codons**. Contour plots showing the length of coding sequence, in triplets, at which the transition from (a) one to two stop codons; (b) two to three stop codons; (c) three to four stop codons; (d) four to five stop codons; and (e) five to six stop codons becomes possible, for values of R from 1 to 100 and values of *k *from 0.1 to 1 (see Equations 1 and 2). Contour lines separate lengths of coding sequence from 0-25 triplets (darkest areas) to 200-225 triplets (lightest areas), in increments of 25 triplets. Transitions to greater numbers of stop codons become increasingly difficult as the number of stop codons increases; the transition from four to five stops is favoured by selection only when the mean coding sequence length is very low (very dark shading over most of the plotted area).

Our model also provides a potential explanation for the lack of known codes with more than four stop codons: for each stepwise change in the number of stop codons from one to five, we can consider the combined costs of premature termination and readthrough. A stop-to-sense reassignment will decrease these combined costs if *C_S+1 _*<*C_S_*. Since *C_S _*= *C*[τ]*_S _*+ *C*[ρ]*_S_*, we can use Equations 1 and 2 and solve for *C_S+1 _*= *C_S _*to give the threshold for the length of coding sequence at which the number of stop codons can increase from *S *to *S*+1. Generally, unless values of *k *are very low - that is, the actual cost of premature termination is substantially reduced by mechanisms such as nonsense-mediated mRNA decay - the transition from four to five stop codons is favoured by selection only when sequence lengths are unfeasibly short; for example, when *k *= 0.1 and *R *= 30, coding sequences would have to be < 167 triplets long (Figure 3).

### A surfeit of stop codons

Every protein necessarily contains only one termination signal; in almost all genomes, stops are used less frequently than any amino acid. Yet, in the canonical genetic code, most amino acids have fewer codons assigned to them than are assigned to stop codons. This disproportionate over-representation is perplexing, especially given that an error which produces a stop codon is likely to be more deleterious than a missense mutation.

We found two potential explanations for this phenomenon in the literature. Firstly, the use of three stop codons may be a maladaptive relic from the origin of life. If the genetic code evolved before accurate nucleic acid replication, three stop codons allow the optimal spacing of open reading frames in primordial DNA or RNA genomes of random sequence [21]. However, this model is in disagreement with other origin-of-life scenarios, including "RNA world", and with the observation that the number of stop codons is surprisingly volatile, with losses of stop codons outnumbering gains [15,17,22]. If multiple stop codons were maladaptive, species with fewer stops would gain a long-term fitness advantage, and it is likely that stop codons would have been reassigned to amino acids in the long time interval between the origin of life and the last common ancestor of all extant organisms.

The second alternative hinges on the ambush hypothesis [23], the idea that selection favours out-of-frame stop codons to minimise the cost of translational frameshift errors. The authors of this hypothesis do consider variant codes, but do not explicitly state that if the same selective forces apply at the level of the code, the ambush hypothesis could provide a counterbalancing benefit of multiple stop codons. However, recent analyses show that codon pairs creating out-of-frame stop codons seem to be generally disfavoured in most sequenced genomes, and particularly in eukaryotes [24]. An increase in out-of-frame stops is therefore not a consistent advantage of using a genetic code with multiple stop codons.

Our analysis may provide part of a much-needed explanation for the apparent profligacy of the universal code and its variants in terms of the number of stop codons. Recent work suggests that variant codes may be far more common than previously thought [25]; we may not have to wait long to obtain sufficient data to allow a rigorous test of the competing theories.

## Conclusions

Codon reassignment imposes a new regime of mutational and transcriptional pressures, and hence new selective pressures on gene length. We find it implausible that the changes in average gene length seen in Figure 2 are selectively neutral, since they represent fundamental pervasive change to whole proteomes. Consequently, a new selective regime imposed by codon reassignment would also make a contribution to the evolutionary success or failure of an organism as a consequence of the effects on fitness arising from changes in gene length. Further work, especially experimental work, will be necessary in order to disentangle cause from effect - note that if codon reassignment and proteome length influence one another, each can be both cause and effect - and to establish the most likely sequence of events involved in stop codon reassignment.

Unfortunately, without values for *R*, which in any case is likely to vary between genes, and *k*, our model - which, like all such models, presents a greatly simplified picture of an extremely complex process - does not allow us predict the optimal number of stop codons in a particular genome. It might be possible to get some indication of plausible values for *R *from, for instance, the number of amino acids that can be added to a protein's C-terminus during purification without affecting its conformation and activity, but since *R *will also vary with the precise downstream sequence (for example, extremely hydrophobic residues might have a greater impact) even this is likely to be largely uninformative. Despite this, our model does allow us to make predictions, such as those tested here, about broad patterns relating to the length of the coding sequence.

## Methods

### Human sequence data

For each of the 28545 protein-coding transcripts annotated in build 36, version 3 of the NCBI RefSeq of the human genome, the coding sequence plus 900 bp immediately downstream of the terminal stop were extracted. Each of these sequences was randomly reordered 1000 times (thus preserving GC content and base composition ratios for each), and for downstream sequence, the position of the first in-frame stop was calculated and compared to the position in the actual data. For both the actual and randomised sequence, where there was no downstream stop in this 900 bp sequence, its position was taken to be at the 300th triplet. The distributions of downstream stop positions in the actual data and in the randomisations were compared using a Kolmogorov-Smirnov test. The same figures were also calculated taking just a single transcript for each of the 22,383 protein-coding genes, with essentially identical results, the average difference per data point between the two approaches being 0.16% (data not shown). Scripts used in the analysis are available on request from the authors.

### Genetic code data

Data on variant genetic codes were obtained from the NCBI (http://www.ncbi.nlm.nih.gov/Taxonomy/Utils/wprintgc.cgi?mode=c); these data are based primarily on reviews by Osawa et al [26] and Jukes & Osawa [27].

### Genome data

Datasets were downloaded from NCBI GenBank's nucleotide database on 16^th ^July 2010 using the following search criteria. In all cases, the taxon name in the Organism field was required. For bacteria and mitochondria, only fully sequenced genomes were compared. "Complete genome" was required in the sequence title, and plasmid sequences were excluded using the NOT option. For eukaryotic nuclear genes, "mitochondrial", "mitochondrion" and "chloroplast" were also excluded; "complete" was required to exclude single exons and partial coding sequences. This criterion also excluded many coding sequences from whole genome sequencing projects, avoiding a comparison between fully sequenced and unsequenced genomes which are likely to differ in genes surveyed.

For mitochondrial datasets excepting Euglenozoa and Haptophyta, "complete genome" plus "mitochondrion" or "mitochondrial" in the title were required. In the Euglenozoa and Haptophyta no fully sequenced mitochondrial genome comparisons were possible; in these cases complete mitochondrial gene sequences were downloaded.

A Perl script then extracted coding sequence co-ordinates, calculated protein length, and attributed lengths to particular genetic codes according to the translation table ascribed in GenBank. Perl scripts and datasets are available on request from the authors.

## Authors' contributions

SLC conceived the project, constructed the model (with RAN), analysed the sequence data and wrote the paper with LJJ. CPL co-conceived the project. JAC, GSE, LJJ and PDP extracted and analysed the sequence and genome data. All authors read and approved the final manuscript.

## Supplementary Material

Additional File 1 **Table S1**. Table S1 details genetic codes, numbers of coding sequences available, mean gene length, and significance of differences in mean gene length, for each taxon shown in Figure 2. Click here for file
