# Supplementary material for: Stops making sense: translational trade-offs and stop codon reassignment
Source: BMC Evol Biol. 2011 Jul 29;11:227. doi: 10.1186/1471-2148-11-227 (PMC3161013; doi:10.1186/1471-2148-11-227)
Supplement: Additional File 1 — Table S1. Table S1 details genetic codes, numbers of coding sequences available, mean gene length, and significance of differences in mean gene length, for each taxon shown in Figure 2. [file 1471-2148-11-227-S1.DOC]

| Taxon | Code Number | # stop codons | Genes | Mean length | Agreement | Significance (t-test) |
| --- | --- | --- | --- | --- | --- | --- |
| Ulvophyceae | 6 | 1 | 67 | 353 |  |  |
|  | 1 (Canonical) | 3 | 84 | 299 |  |  |
|  | 11 | 3 | 132 | 232 | Yes | NS |
| Intramacronucleata | 6 | 1 | 2191 | 482 |  |  |
|  | 10 | 2 | 184 | 393 | Yes | NS |
|  | 1 (Canonical) | 3 | 15 | 382 | Yes | NS |
| Postciliodesmatophora | 6 | 1 | 2 | 406 |  |  |
|  | 15 | 2 | 11 | 297 | Yes | NS |
| Diplomonads | 6 | 1 | 32 | 481 |  |  |
|  | 1 (Canonical) | 3 | 6936 | 434 | Yes | NS |
| Mollicutes | 4 | 2 | 37524 | 369 |  |  |
|  | 11 | 3 | 9293 | 285 |  |  |
|  | 1 (Canonical) | 3 | 3 | 152 | Yes | ** p<0.001 |
| Rhizobiales | 4 | 2 | 338 | 257 |  |  |
|  | 11 | 3 | 19441 | 313 | No | ** p<0.001 |
| Chordata mitochondrial | 13 | 2 | 61 | 284 |  |  |
|  | 5 | 2 | 52 | 279 |  |  |
|  | 2 | 4 | 51414 | 291 | No | NS |
| Fungi mitochondrial | 3 | 2 | 141 | 355 |  |  |
|  | 4 | 2 | 1209 | 293 |  |  |
|  | 16 | 2 | 74 | 284 |  |  |
|  | 1 (Canonical) | 3 | 50 | 280 | Yes | * p<0.05 |
| Rhodophyta mitochondrial | 4 | 2 | 90 | 252 |  |  |
|  | 1 (Canonical) | 3 | 34 | 246 |  |  |
|  | 11 | 3 | 394 | 237 | Yes | NS |
| Chlorophyta mitochondrial | 4 | 2 | 22 | 331 |  |  |
|  | 22 | 3 | 38 | 296 |  |  |
|  | 1 (Canonical) | 3 | 699 | 266 |  |  |
|  | 11 | 3 | 121 | 247 | Yes | * p<0.05 |
| Stramenopiles mitochondrial | 4 | 2 | 69 | 309 |  |  |
|  | 1 (Canonical) | 3 | 1023 | 237 | Yes | * p<0.05 |
| Haptophyta mitochondrial | 4 | 2 | 33 | 104 |  |  |
|  | 1 (Canonical) | 3 | 6 | 279 | No | NS |
| Euglenozoa mitochondrial | 4 | 2 | 23 | 445 |  |  |
|  | 1 (Canonical) | 3 | 26 | 361 | Yes | NS |
